# An Autocrine TNFα–Tumor Necrosis Factor Receptor 2 Loop Promotes Epigenetic Effects Inducing Human Treg Stability *In Vitro*

**DOI:** 10.3389/fimmu.2018.00573

**Published:** 2018-03-21

**Authors:** Paulo C. M. Urbano, Hans J. P. M. Koenen, Irma Joosten, Xuehui He

**Affiliations:** ^1^Laboratory of Medical Immunology, Department of Laboratory Medicine, Radboud University Medical Center, Nijmegen, Netherlands; ^2^College of Computer Science, Qinghai Normal University, Xining, China

**Keywords:** regulatory T cells, tumor necrosis factor receptor 2, TNFα, rapamycin, tumor necrosis factor receptor 2 agonist antibody, Treg stability

## Abstract

A crucial issue for Treg-based immunotherapy is to maintain a *bona fide* Treg phenotype as well as suppressive function during and after *ex vivo* expansion. Several strategies have been applied to harness Treg lineage stability. For instance, CD28 superagonist stimulation *in vitro*, in the absence of CD3 ligation, is more efficient in promoting Treg proliferation, and prevention of pro-inflammatory cytokine expression, such as IL-17, as compared to CD3/CD28-stimulated Treg. Addition of the mTOR inhibitor rapamycin to Treg cultures enhances FOXP3 expression and Treg stability, but does impair proliferative capacity. A tumor necrosis factor receptor 2 (TNFR2) agonist antibody was recently shown to favor homogenous expansion of Treg *in vitro*. Combined stimulation with rapamycin and TNFR2 agonist antibody enhanced hypo-methylation of the *FOXP3* gene, and thus promoting Treg stability. To further explore the underlying mechanisms of rapamycin and TNFR2 agonist-mediated Treg stability, we here stimulated FACS-sorted human Treg with a CD28 superagonist, in the presence of rapamycin and a TNFR2 agonist. Phenotypic analysis of expanded Treg revealed an autocrine loop of TNFα–TNFR2 underlying the maintenance of Treg stability *in vitro*. Addition of rapamycin to CD28 superagonist-stimulated Treg led to a high expression of TNFR2, the main TNFR expressed on Treg, and additional stimulation with a TNFR2 agonist enhanced the production of soluble as well as membrane-bound TNFα. Moreover, our data showed that the expression of histone methyltransferase EZH2, a crucial epigenetic modulator for potent Treg suppressor function, was enhanced upon stimulation with CD28 superagonist. Interestingly, rapamycin seemed to downregulate CD28 superagonist-induced EZH2 expression, which could be rescued by the additional addition of TNFR2 agonist antibody. This process appeared TNFα-dependent manner, since depletion of TNFα using Etanercept inhibited EZH2 expression. To summarize, we propose that an autocrine TNFα–TNFR2 loop plays an important role in endorsing Treg stability.

## Introduction

CD4^+^FOXP3^+^ regulatory T cells (Treg) inhibit autoreactive effector T cells (Teff) and are important for immune homeostasis. The absence of Treg leads to lethal autoimmune disease in mice and humans, thereby highlighting their critical role in preventing autoimmunity (1). Notwithstanding the first successes of translation of Treg-based cell therapy into the clinic, a critical concern in utilizing Treg is their stability. Treg lineage stability is defined by a stable expression of the transcription factor FOXP3, a highly demethylated Treg-specific demethylation region (TSDR), potent suppressive capacity and lack of pro-inflammatory cytokine production (2, 3). For the efficacy of Treg-based immunotherapy, the development of optimal *ex vivo* expansion protocols that yield high numbers of stable Treg is a prerequisite. Standard expansion protocols using anti-CD3/anti-CD28 mAb-coated microbeads plus exogenous rhIL-2 not only lead to high cell yields (4, 5) but also reveal Treg plasticity, whereby Treg loose FOXP3 and start producing IL-17A and IFNγ (6–8). Stimulating Treg with an anti-CD28 superagonist antibody (CD28-SA) results in efficient Treg expansion and reduced pro-inflammatory cytokine production *in vitro* (9). Since Treg are less susceptible to rapamycin-mediated inhibition of cell proliferation as compared to non-Treg cells, this mTOR inhibitor is often added to Treg expansion cultures to increase the purity of the final cell product (10–16). However, rapamycin does limit Treg growth both *in vitro* and *in vivo* (17, 18). It is of interest to note that the combined addition of a tumor necrosis factor receptor 2 (TNFR2) agonistic monoclonal antibody and rapamycin not only rescues rapamycin-mediated inhibition of Treg proliferation but also leads to a highly homogenous Treg phenotype as well as a stable suppressive function upon expansion (19, 20).

TNFα is initially expressed on cell surface as a membrane-bound TNFα (mTNFα), which can be cleaved by a metalloprotease TNF-alpha converting enzyme (TACE) to generate soluble TNFα (sTNFα) (21). Both sTNFα and mTNFα bind to TNFR2, but only mTNFα is capable to fully activate TNFR2 downstream signal events including NFkB pathway, which is involved in cytokine storm, cell survival and proliferation (22, 23). TNFR2 is constitutively expressed on both murine and human Treg, and TNFR2^+^ Treg are the most suppressive Treg subpopulation (24–27). The TNFα–TNFR2 interaction is required for Treg mediated suppression in a mouse model of autoimmune-mediated colitis (28, 29). Several studies demonstrated that sTNFα preserved or even increased FOXP3 expression, as well as Treg suppressive capacity in both mice and humans (19, 25, 30, 31). But anti-TNF therapy of patients with active rheumatoid arthritis restored FOXP3 expression as well as suppressive function (32). Notably, the high serum levels of TNFα were associated with increased peripheral Treg numbers in patients with colorectal cancer and hepatocellular carcinoma, where blockade of TNFα/TNFR2 signals inhibited Treg cell expansion and benefited cancer therapy (33), thereby indicating that TNFα is capable of mediating Treg expansion.

Treg lineage stability is ultimately maintained by sustained expression of FOXP3 and Treg-specific epigenetic modification patterns (34). In response to inflammatory cues, FOXP3 recruits the histone methyltransferase EZH2 at the FOXP3-bound loci and selectively deposits the transcriptional suppression mark trimethylation of histone H3 at lysine 27 (H3K27me3) (35). In mice, it was shown that EZH2 expression was induced in a CD28-dependent manner and the mutant mice bearing Treg-specifically depletion of EZH2 developed fetal multi-organ autoimmunity with excessive T cell activation (36). Of note, EZH2-deficient FOXP3^+^ murine T cells secreted pro-inflammatory cytokines (37). It is not yet clear whether human Treg show similar EZH2 expression metrics. Microarray analysis of human naïve T cells revealed that *EZH2* gene was the most highly induced CD28-dependent chromatin modifier (36).

Having previously established that a CD28 superagonist mAb (CD28-SA) acts as a very effective stimulus to support efficient Treg expansion (9), and that the combined use of rapamycin and TNFR2 agonist enhanced the demethylation of TSDR, thus harnessing Treg stability (20), we further explored Treg *ex vivo* stimulation and maintenance of stability by combining CD28 superagonist mAb, rapamycin and TNFR2 agonist mAb. We found that the harnessing effect of rapamycin and TNFR2 agonist on Treg stability was achieved through an autocrine loop of TNFα *via* TNFR2, whereby rapamycin enhanced TNFR2 expression and TNFR2 agonist increased the production of TNFα. Moreover, our data demonstrated that, similar to murine Treg, the histone methyltransferase EZH2 was induced in human Treg upon CD28 superagonist stimulation. Intriguingly, the combined addition of rapamycin and TNFR2 agonist maintained EZH2 expression in a TNFα-dependent manner.

## Material and Methods

### Isolation of Human Treg

Peripheral blood mononuclear cells were isolated by density gradient centrifugation (Lymphoprep, Nycomed Pharma AS, Oslo, Norway) of buffy coats that were purchased from Sanquin blood bank (Region South-East, Netherlands). All donors gave written informed consent for the use of these buffy coats for scientific research purposes, and according to Dutch law. CD4^+^ T cells were enriched using the RosetteSep™ human CD4^+^ T cell enrichment cocktail and processed according to manufacturer’s recommendations (StemCell Technologies, Vancouver, BC, Canada). This typically resulted in a >95% purified CD4^+^ T cell population in the absence of CD8^+^ cells. To obtain high purity Treg, subsequent FACS sorting of CD4^+^CD25^high^ Treg was performed using a BD FACSAria cell sorter (BD Biosciences, Erembodegem, Belgium) after labeling CD4^+^ cells with CD25/Pe-Cy7 (M-A251; BD Biosciences).

### Treg Cell Culture

FACS-sorted CD4^+^CD25^high^ Treg were cultured for 7 days with IL-2 (200 U/mL) containing medium alone as non-stimulated control, or together with different combinations of CD28 superagonist (CD28-SA, 1 µg/mL, Clone ANC28.1/5D10, Cat# 177-820, preservative free; Ancell, Bayport, MN, USA), rapamycin (Rap, 1 µM, Sigma-Aldrich, St. Louis, MO, USA), and TNFR2 agonist mAb (2.5 µg/mL, Clone MR2-1, Hycult, Netherlands). Exogenous recombinant human (rh) TNFα (50 ng/mL, R&D, Minneapolis, MN, USA) was used to replace TNFR2 agonist where indicated. Etanercept (10 µg/mL, ETN-Enbrel^®^, Pfizer) was added to cell culture for the depletion of TNFα. Cells were harvested at day 7 of culture for phenotypic analysis, and culture supernatants were collected and stored for the subsequent cytokine analysis.

### Flow Cytometry and Antibodies

Cells were phenotypically analyzed using a multicolor flow cytometer Navios (Beckman Coulter, Mijdrecht, Netherlands). The following conjugated mAb were used: CD25/Pe-Cy7 (M-A251), HLA-DR/FITC (L243) (both from BD Bioscience); TIGIT/PE (MBSA43, eBioscience, Vienna, Austria), CD3/ECD (UCHT1), CD4/PE-Cy5.5 (1388.2), CD8/APC-AF700 (B9.11) (all from Beckman Coulter), TNFR2/APC (#22235; R&D), and Fixable Viability Dye eFluor780 (eBioscience). To detect the expression of mTNFα, cells were first stained with biotin-labelled Infliximab followed with APC-conjugated streptavidin (eBioscience). For intracellular staining, EZH2/PE (11/EZH2, BD Bioscience), FOXP3/eFluor 450 (PCH101), and Helios/AlexFluor 647 (22F6) (both from eBioscience) were used after fix-perm-treatment of cells, according to the manufacturer’s instructions. Isotype matched control antibodies were used to define marker settings. Data were analyzed using the software Kaluza (Beckman Coulter).

### Cytokine Detection Assay

IL-17A, IFNγ, and TNFα were determined in the culture supernatants using Luminex cytokine assays (Invitrogen), according to the manufacturer’s instructions. The lower levels of detectable cytokines were IL-17A (2 pg/mL), IFNγ (2.3 pg/mL), and TNFα (2.3 pg/mL).

### Coculture Suppression Assays

FACS-sorted Treg cells were cultured under the stimulation conditions described above. Thereafter, cultured Treg were collected at day 7 of culture, washed, and added at different ratios to CFSE-labeled CD4^+^CD25^−^ responder T cells (Tresp). Coculture mixture was stimulated with anti-CD3/anti-CD28 mAb-coated microbeads at a bead-to-cell ratio of 1:5 for 3 days before analyzing the dilution of CFSE using flow cytometry.

### Quantitative Real-time PCR (RT-qPCR)

Total RNA was extracted by using the RNeasy Plus Micro kit (Qiagen, Hilden, Germany) followed by cDNA synthesis using the SuperScript III First-Strand Synthesis System and Oligo(dT)20 primers (Thermo Fisher Scientific, Waltham, MA, USA). Taqman gene expression assays were purchased from Thermo Fisher Scientific (see Table S1 in Supplementary Material). RT-qPCR cycle values (CT) obtained for specific mRNA expression in each sample were normalized to the CT values of the housekeeping gene HPRT1 (endogenous control). The relative mRNA expression of gene interested was calculated using 2^−ΔCT^ formula.

### Statistics

Statistical analysis was performed using the GraphPad Prism software version 5.0 (GraphPad Software Inc., San Diego, CA, USA). Statistical differences were calculated using the Wilcoxon matched pairs signed rank test, or the non-parametric Friedman test or Kruskal–Wallis test plus Dunn’s *post hoc* test for multiple comparisons, where applicable. Differences were considered statistically significant at *p < 0.05, **p < 0.01, or ***p < 0.001.

## Results

### Rapamycin Increases the Expression of TNFR2 on CD28 Superagonist-Stimulated Treg

**Figure 1:**
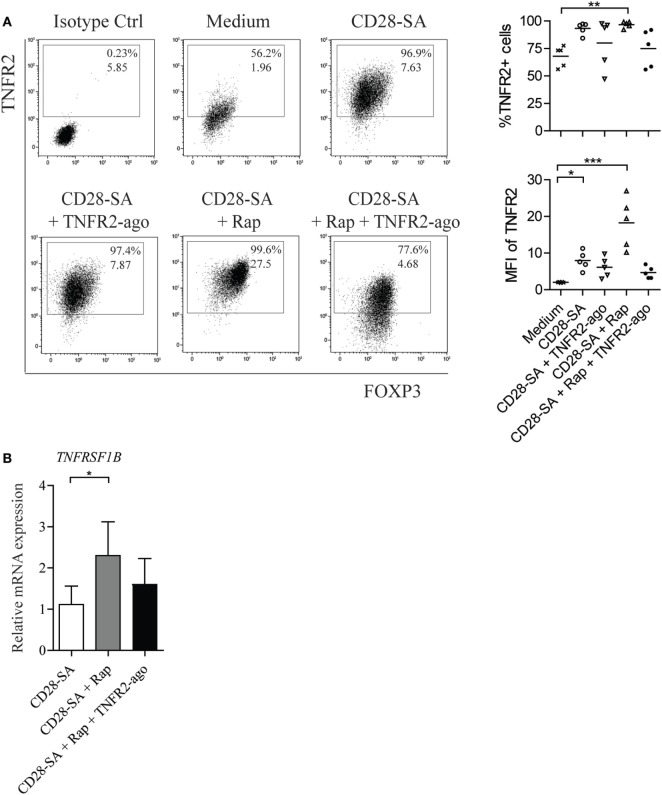
Rapamycin increases TNFR2 expression on human Treg following the stimulation with CD28 superagonist. Flow cytometry of TNFR2 expression on human Treg that were expanded for 7 days under the indicated conditions (legends): non-stimulated medium control (Medium), stimulation with CD28 superagonist mAb (CD28-SA) with and without rapamycin in the absence or presence of TNFR2 agonist mAb (CD28-SA, CD28-SA + TNFR2-ago, CD28-SA + Rap, CD28-SA + Rap + TNFR2-ago). **(A)** Dot plots show TNFR2 vs. FOXP3 expression of one representative donor. Cumulative data showing the percentage as well as the median fluorescent intensity (MFI) of TNFR2 expression on Treg cultured under the conditions as indicated on the *X*-axis. *N* = 5. Lines show the mean values. **(B)** Relative mRNA expression of *TNFRSF1B* in Treg stimulated with the conditions as described on the *X*-axis. *N* = 4. All data are shown as mean ± SEM. Friedman with Dunn’s *post hoc* test was used for statistical analysis. Asterisks indicate significant differences (*p < 0.05, **p < 0.01, or ***p < 0.001).

Tumor necrosis factor receptor 2 is known to be crucial for phenotypic and functional stability of Treg, especially in an inflammatory environment (29). We thus started off by examining the expression level of TNFR2 on human FACS-sorted CD4^+^CD25^high^ Treg stimulated with a CD28 superagonist mAb (CD28-SA) in the presence or absence of Rap and/or TNFR2 agonist. Treg cultured in IL-2 containing medium alone were used as non-stimulated control, and the expression of TNFR2 was determined by flow cytometry at day 7 of culture. In the absence of CD28-SA stimulation, TNFR2 agonist itself neither revealed a potential cytotoxic effect on cultured Treg nor the regulation of TNFR2 expression as compared to Treg cultured under the medium control condition (Figure S1A–B in Supplementary Material). Stimulation of Treg using CD28-SA significantly enhanced the expression of TNFR2 (92.5 ± 2.9 vs. 70.5 ± 3.1% for medium control, *p* < 0.05), while the addition of Rap to CD28-SA stimulated Treg resulted in the highest expression level of TNFR2, both in frequency (95.5 ± 1.7%; *p* < 0.01) and in median fluorescence intensity [median fluorescent intensity (MFI), 18.9 ± 3.9 vs. 2.1 ± 0.2 for medium control; *p* < 0.001] (Figure 1A). The addition of TNFR2 agonist to CD28-SA stimulated Treg hardly affected TNFR2 expression as compared to CD28-SA (MFI, 6.10 ± 1.2 vs. 7.93 ± 1.1, *p* > 0.05). Surprisingly, Treg cultured with the triple combination of CD28-SA + Rap + TNFR2 agonist expressed a similar level of TNFR2 (77.9 ± 6.3%) as that observed for control Treg (70.5 ± 3.1%, *p* > 0.05) (Figure 1A). The potential cytotoxic effect of TNFR2 agonist on cultured Treg is unlikely as we observed similar cell viability under all conditions tested (Figure S2 in Supplementary Material). Interference of the TNFR2 agonist with the subsequent detection of TNFR2 in this case was also not likely, as we selectively chose an APC-conjugated anti-TNFR2 mAb (Clone #22235) derived from a different clone than the TNFR2 agonist (Clone MR2-1). FITC-conjugated TNFR2 mAb derived from the same clone MR2-1 as TNFR2 agonist used in Treg culture failed to detect any expression of TNFR2, whereas APC-conjugated TNFR2 did (Figure S3 in Supplementary Material). Instead, we propose that binding of TNFR2 agonist might have caused the internalization of the TNFR2–ligand complex (38), leading to lower levels of detection. The rapamycin enhanced TNFR2 expression was also reflected at mRNA level since the highest *TNFRSF1B* (TNFR2) mRNA was observed under the condition of CD28-SA + Rap (Figure 1B). Taken together, the data suggest that Rap increases TNFR2 expression on Treg following cell stimulation.

### The Addition of Rap and TNFR2 Agonist to CD28 Superagonist-Stimulated Treg Initiates an Autocrine TNFα–TNFR2 Loop

**Figure 2:**
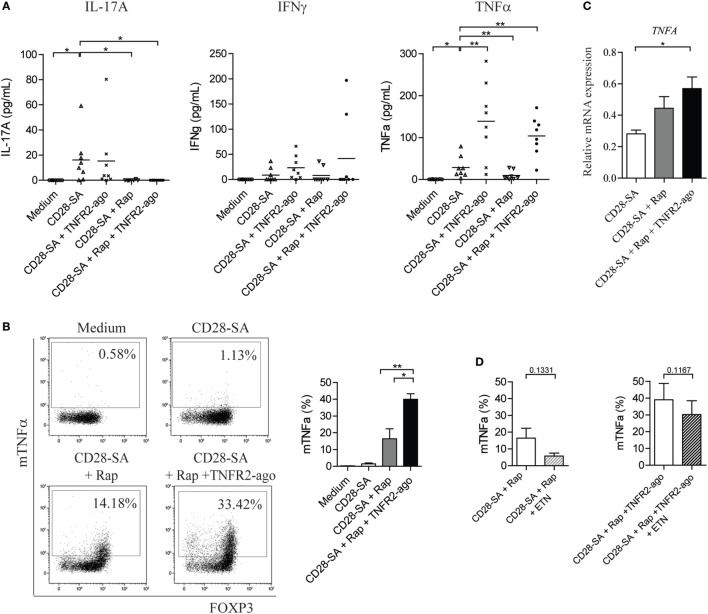
Addition of rapamycin and TNFR2 agonist to CD28-superagonist stimulated Treg initiates an autocrine TNFα–TNFR2 loop. FACS-sorted human Treg were stimulated for 7 days as indicated on the *X*-axis; the culture supernatants were collected at day 7 and the presence of cytokines were determined using Luminex. **(A)** Cumulative data showing the amount of IL-17A, IFNγ, and TNFα produced by Treg cultured as described on the *X*-axis. *N* = 8. **(B)** Flow cytometry analysis of membrane-bound TNFα (mTNFα) as well as FOXP3 expression at day 7 of culture. Dot plots showing one representative donor. Graph shows the cumulative data. *N* = 4. Percentage of mTNFα-positive cells is indicated in the dot plots. **(C)** Relative mRNA expression of *TNFA* in Treg stimulated with the conditions as described on the *X*-axis. *N* = 4. **(D)** FACS-sorted human Treg were stimulated with CD28-SA + Rap or CD28-SA + Rap + TNFR2 agonist in the presence or absence of the TNFα-blocking agent Etanercept (ETN). Cumulative data showing the percentage of mTNFα^+^ cells at day 7 of culture. *N* = 3. All data are shown as mean ± SEM. Friedman with Dunn’s *post hoc* test were used for statistical analysis. Asterisks indicate significant differences (*p < 0.05, **p < 0.01).

Loss of Treg stability implies that Treg acquire the capacity to produce effector cytokines upon stimulation. We therefore measured the amount of IL-17A, IFNγ, and TNFα in the culture supernatants of Treg that were stimulated under distinct conditions. Neither Treg cultured in medium control condition nor that cultured in the presence of TNFR2 agonist produced any cytokines (Figure S1C in Supplementary Material), whereas upon CD28-SA stimulation, Treg started to produce low, but detectable amounts of IL-17A, IFNγ, and TNFα. The addition of Rap to the culture prevented CD28-SA stimulated Treg to produce IL-17A (0.1 ± 0.1 vs. 16.2 pg/mL ± 6.7, *p* < 0.05), as well as TNFα (9.7 ± 3.9 vs. 28.6 pg/mL ± 9.1, *p* < 0.01), but it marginally affected IFNγ production (Figure 2A). The addition of TNFR2 agonist to the culture minimally regulated CD28-SA induced IL-17A and IFNγ production, whereas it increased the amount of TNFα (139.0 ± 32.85 vs. 28.62 pg/mL ± 9.1, *p* < 0.05). Notably, adding TNFR2 agonist to Rap treated CD28-SA stimulated Treg resulted in a similar high amount of TNFα (103.7 pg/mL ± 16.4) as that of Treg stimulated with CD28-SA + TNFR2 agonist (Figure 2A).

Soluble TNFα is derived from its precursor mTNFα, whereby mTNFα is cleaved by the TNFα-converting enzyme TACE to release its extracellular C-terminal portion (21). To test whether the enhanced soluble TNFα production was due to the increased conversion from its precursor, we analyzed the expression of mTNFα on Treg cultured under different combinations of CD28-SA, Rap, and TNFR2 agonist. At day 7 of culture, few mTNFα^+^ cells were detected on CD28-SA stimulated Treg (1.5 ± 0.5%), whereas addition of Rap enhanced mTNFα expression (16.4 ± 5.9%). However, triple stimulation with CD28-SA + Rap + TNFR2 agonist further promoted the frequency of mTNFα^+^ cells (40.0 ± 3.3%, *p* < 0.05, Figure 2B). Similarly, the highest expression of *TNFRA* (TNFα) mRNA was observed under triple stimulation with CD28-SA + Rap + TNFR2 agonist (Figure 2C). This data indicates that the combined addition of Rap and TNFR2 agonist to CD28-SA stimulated Treg did increase their capacity to produce more TNFα. So, Rap treatment increased the expression of TNFR2 on CD28-SA stimulated Treg cells, while the additional treatment with a TNFR2 agonist significantly enhanced TNFα production. This might well result in an autocrine loop of TNFα *via* TNFR2, thus leading to stabilization of the Treg phenotype.

To find further support for this autocrine TNFα–TNFR2 loop, we depleted TNFα by using Etanercept. As shown in Figure 2D, regardless of the stimulation condition used, extra addition of Etanercept resulted in decreased mTNFα expression, albeit not statistically significant. Taken together, the data show that there is a positive feedback loop in the regulation of TNFα cytokine production upon TNFα–TNFR2 interaction.

### The TNFα–TNFR2 Interaction Is Required for a Homogenous Treg Phenotype

**Figure 3:**
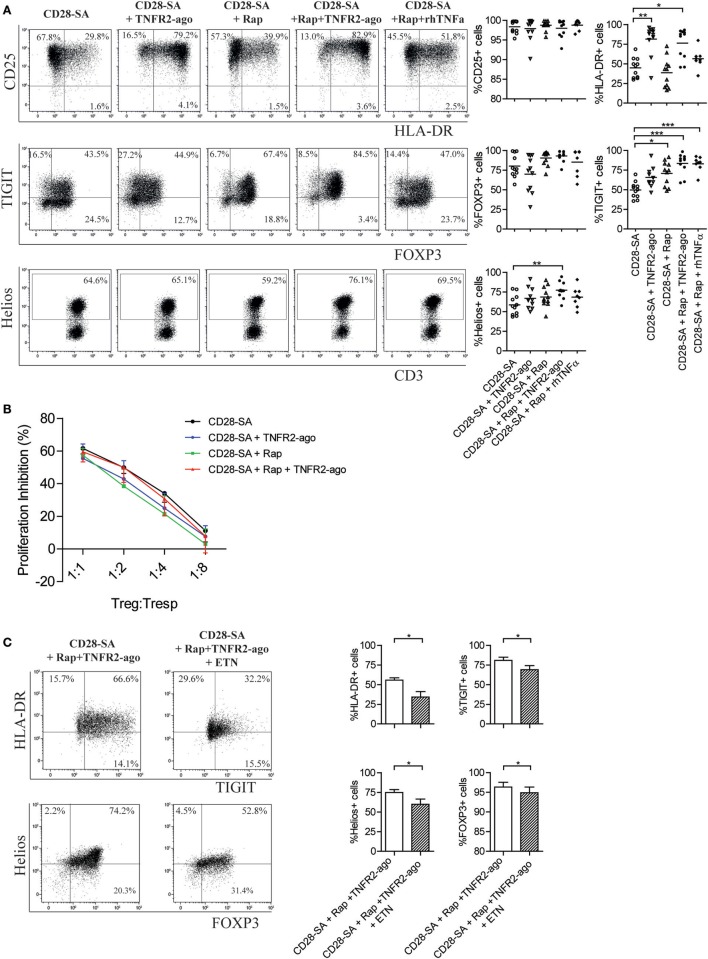
Addition of rapamycin and TNFR2 agonist to CD28-superagonist stimulated Treg cultures leads to a homogenous Treg phenotype that is dependent on the interaction of TNFα–TNFR2. **(A)** Flow cytometry of CD25, HLA-DR, TIGIT, FOXP3, and Helios expression on CD28-SA stimulated Treg that were additionally cultured with Rapamycin (Rap) with or without TNFR2 agonist or soluble rhTNFα as indicated. Dot plots show representative result of one blood donor. Cumulative data are given in the graphs. *N* = 8–11. Lines show the mean values. **(B)** Treg cultured under the indicated conditions (legend) were harvested at day 7 of culture, washed, allowed to recuperate, and analyzed for their suppressive capacity in a CFSE-based coculture suppression assay. *N* = 4. Friedman with Dunn’s *post hoc* test were used for statistical analysis. **(C)** FACS-sorted human Treg were stimulated with CD28-SA + Rap + TNFR2 agonist in the presence or absence of the TNFα-blocking agent Etanercept (ETN). Dot plots showing TIGIT vs. HLA-DR, and Helios vs. FOXP3 expression of one representative experiment. Cumulative data are shown in the graph. *N* = 7. Numbers in dot plots show the percentage of positive cells. All data are shown as mean ± SEM. Friedman with Dunn’s *post hoc* test was used for statistical analysis. Asterisks indicate significant differences (*p < 0.05, **p < 0.01, or ***p < 0.001).

Potent Treg function is associated with high expression of specific cell markers, including Treg lineage transcription factor FOXP3, Helios, and the co-inhibitory receptor TIGIT (39–41). We thus performed phenotypic analysis of Treg that were cultured for 7 days under distinct stimulatory conditions. TNFR2 agonist itself hardly influenced the Treg phenotype (Figure S1D in Supplementary Material). When cells were stimulated with CD28-SA, the addition of Rap preserved or even slightly increased the expression of CD25 as well as FOXP3, Helios, and TIGIT (Figure 3A). Intriguingly, the addition of TNFR2 agonist to CD28-SA stimulated Treg clearly enhanced the expression of HLA-DR (81.5 ± 5.9 vs. 44.8 ± 3.9%, *p* < 0.01) while it hardly regulated other markers tested (Figure 3A). The combined addition of Rap and TNFR2 agonist to CD28-SA stimulated Treg significantly enhanced the frequency of the HLA-DR, TIGIT and Helios positive fractions (Figure 3A, *p* < 0.01), and preserved the high expression of FOXP3. Of note, when exogenous soluble rhTNFα was used instead of the TNFR2 agonist, we observed a similar expression of CD25, FOXP3, TIGIT, and Helios, but not of HLA-DR, which was only enhanced by the presence of the TNFR2 agonist (Figure 3A). Treg stimulated with CD28-SA + Rap + TNFR2 agonist were highly suppressive, as determined in *in vitro* suppression assays. We did not observe significant suppressive advantages as compared to the Treg that were cultured under the other stimulatory culture conditions (Figure 3B). Interestingly, depletion of TNFα under triple stimulation with CD28-SA + Rap + TNFR2 agonist significantly downregulated the expression of HLA-DR, TIGIT, Helios, and FOXP3 (Figure 3C). These data further support the notion of an autocrine TNFα–TNFR2 feedback loop that promotes a homogeneous Treg population upon activation, whereby Rap enhances TNFR2 expression and TNFR2 agonist stimulation increases TNFα production.

### The Addition of Rap and TNFR2 Agonist to CD28 Superagonist-Stimulated Treg Leads to Activation of NFκB Signal Pathway

**Figure 4:**
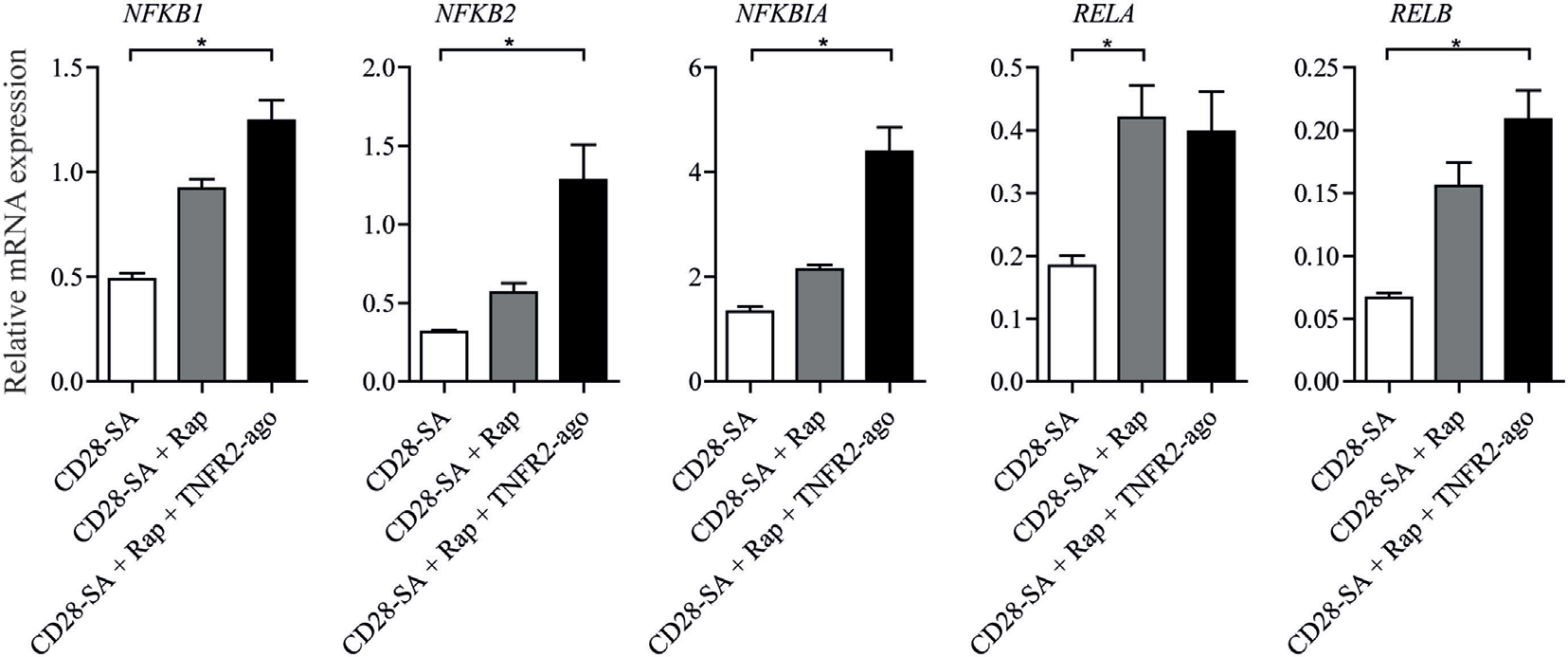
Addition of rapamycin and TNFR2 agonist to CD28-superagonist stimulated Treg cultures results in the activation of the NFκB pathway. FACS-sorted Treg cells were stimulated using CD28-SA, CD28-SA + Rap, or CD28-SA + Rap + TNFR2 agonist for 4 days. The mRNA expression of NFκB family of members was analyzed using RT-qPCR. All data are shown as mean ± SEM. *N* = 4. Friedman with Dunn’s *post hoc* test was used for statistical analysis. Asterisks indicate significant differences (*p < 0.05).

To test the potential involved downstream signal pathways that were induced by triple stimulation with CD28-SA + Rap + TNFR2 agonist, we focused on NFκB pathway target genes using RT-qPCR analysis. Treg stimulated with CD28-SA or CD28-SA + Rap were also included. As shown in Figure 4, the addition of Rap to CD28-SA stimulated Treg led to the enhanced *RELA* (RelA) mRNA expression, whereas the combined addition of Rap and TNFR2 agonist significantly increased the NFκB pathway gene expression including *NFKB1* (NFκB1/p65), *NFKB2* (NFκB2/p50), *NFKBIA* (IkBα), and *RELB* (RelB). The data suggest that the activation of the NFκB pathway underlies the enhanced Treg stability mediated by the autocrine TNFα–TNFR2 loop.

### TNFα–TNFR2 Signaling Regulates the Expression of Histone Methyltransferase EZH2

**Figure 5:**
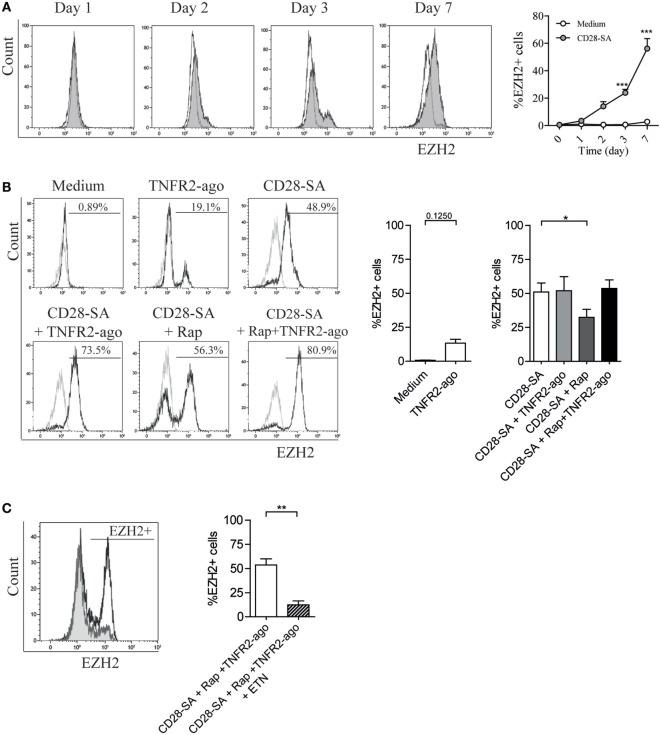
CD28 superagonist induces EZH2 expression in human Treg and addition of rapamycin downregulates EZH2 frequency which can be rescued by TNFR2 agonist in a TNFα-dependent manner. Flow cytometry of EZH2 expression in FACS-sorted human Treg that were cultured for 7 days in medium (Medium) or stimulated with CD28 superagonist mAb (CD28-SA) with or without Rapamycin (Rap) in the absence or presence of TNFR2 agonist mAb, or the TNFα blocker Etanercept (ETN). **(A)** Representative experiment showing EZH2 expression at different days in time (indicated at the top). Graph shows cumulative data. *N* = 3. Open circle: medium control; shaded circle, CD28-SA stimulation. **(B)** Representative overlay histograms showing EZH2 expression at day 7 of the culture. Graph shows cumulative data. *N* = 12. Numbers in the overlay histograms show the percentage of EZH2-positive cells. Gray line: isotype control; black line, Treg expanded under conditions where described at the top. *N* = 4 for the medium control and TNFR2 agonist conditions, *N* = 8 for CD28-SA + TNFR2 agonist condition, and *N* = 12 for other conditions. Kruskal–Wallis with Dunn’s *post hoc* test was used for statistical analysis. **(C)** Representative overlay histogram showing EZH2 expression in Treg that were stimulated with CD28-SA + Rap + TNFR2 agonist together with or without ETN. *N* = 7. Graph shows the cumulative data. Wilcoxon matched pairs signed rank test was used for statistical analysis. All data are shown as mean ± SEM. Asterisks indicate significant differences (*p < 0.05, **p < 0.01, or ***p < 0.001).

Recently, CD28-dependent induction of histone methyltrasferase EZH2 was reported in murine Treg (36). In the same study, EZH2 was shown to be crucial for Treg lineage stability following cell activation. Here, we first performed a time kinetic analysis of EZH2 expression in human Treg stimulated with CD28 superagonist. From day 2 of culture, enhanced EZH2 expression was detected, and the highest frequency of EZH2-positive Treg was observed at day 7 (47.6 ± 5.5 vs. 2.9 ± 1.3% for medium control, *p* < 0.001) (Figure 5A). Thereafter, we focused on day 7 to analyze the effect of Rap and/or TNFR2 agonist on the expression of EZH2. As shown in Figure 5B, TNFR2 agonist itself slightly enhanced the expression of EZH2 (13.4 ± 2.8 vs. 2.9 ± 1.0% for medium control group, *p* = 0.1250). When Treg were stimulated with CD28-SA, addition of TNFR2 agonist to the culture minimally affected EZH2 expression (51.6 ± 10.3 vs. 51.0 ± 6.6% for CD28-SA condition), whereas addition of Rap decreased EZH2 expression (32.3 ± 5.9%) when compared to CD28-SA condition (*p* < 0.05). Of note, the combined addition of Rap and TNFR2 agonist resulted in a similar frequency of EZH2-positive cells (53.6 ± 6.3%) as compared to CD28-SA condition, suggesting that the presence of TNFR2 agonist could rescue Rap-mediated downregulation of EZH2. Thus, TNFR2 agonist induced signals were positively involved in the regulation of EZH2 expression. Indeed, when TNFα was depleted by adding Etanercept to triple stimulated (CD28-SA + Rap + TNFR2 agonist) Treg, the frequency of EZH2-positive cells was significantly decreased (Figure 5C). Altogether, the data indicate that EZH2 expression is modulated by TNFα–TNFR2-mediated pathways.

## Discussion

The limited number of circulating Treg and the instability and plasticity of Treg function are main issues that hamper successful application of Treg for clinical cell-based immunotherapy. In the past decades, several interventions have been used to optimize Treg *ex vivo* expansion protocols that not only maximize Treg proliferation but also maintain their potent suppressive function. Standard Treg expansion protocols include anti-CD3 and anti-CD28 mAb together with the exogenous addition of rhIL-2 cytokines (4). In the absence of anti-CD3, single stimulation of human Treg with a CD28 superagonist induces polyclonal expansion of Treg with enhanced Treg stability (9). The mTOR inhibitor rapamycin enhances FOXP3 expression, preserves Treg stability, and increases Treg suppressor capacity *in vitro* as well as *in vivo*, but rapamycin also inhibited Treg cell proliferation (15, 17, 42, 43). Previously, we showed that the combined addition of rapamycin and TNFR2 agonist to Treg cell culture facilitates *ex vivo* expansion of Treg (20). In this study, we found that rapamycin enhanced the expression of TNFR2 on activated Treg and that the additional supplementation of a TNFR2 agonist enhanced the production of TNFα. This resulted in a positive autocrine feedback loop of TNFα–TNFR2 signaling that promotes Treg stability as indicated by the high expression of FOXP3, Helios, and EZH2, and the low production of the pro-inflammatory cytokine IL-17A. Despite this increased expression of FOXP3, Helios, and EZH2, we did not observe an increase in suppressor potential. This is remarkable, since our group and others have demonstrated before that TNFα–TNFR2 stimulation increases Treg function in both humans and mice (19, 20, 44). Previously, we reported that Treg stimulated with CD3/CD28-microbeads + Rap + TNFR2 agonist hardly produced IL-17A and IFNγ, and these cells revealed superior suppressive activity at a Treg:Tresp ratio of 1:8 as compared to CD3/CD28 or CD3/CD28 + Rap-stimulated Treg (20). In our current study, CD28-SA + Rap + TNFR2 agonist-treated Treg showed similar suppressive capacity as Treg treated with CD28SA, CD28SA + Rap, or CD28SA + TNFR2 agonist. It seems that TNFR2-mediated signals somehow interact with T cell receptor/CD3 induced downstream targets and promote Treg suppressor function. Furthermore, Treg display their immunosuppressive function *via* controlling T cell proliferation and cytokine production, as well as regulating the stimulatory capacity of antigen presenting cells. Especially, inhibition of T cell effector function can occur independently of suppression of proliferation (45, 46). Loss of Treg lineage commitment is often reflected by the decreased expression of Treg markers on their progenies, which mostly occurs following several rounds of stimulation.

Tumor necrosis factor receptor 2 plays a crucial role in Treg cell biology. Both human and murine Treg constitutively express high levels of TNFR2 as opposed to non-Treg cells. The interaction of TNFα–TNFR2 promoted both Treg proliferation and their suppressor capacity (25, 47). Stimulation of TNFR2 using a TNFR2 agonist antibody resulted in a homogenous expansion of human Treg (19, 20). Interestingly, we here demonstrate that rapamycin enhanced the expression of TNFR2 on activated human Treg, whereas it inhibited TNFα cytokine production. When TNFR2 agonist was added to rapamycin-treated Treg cell cultures, we found the preferential stimulation of Treg with high expression levels of HLA-DR, FOXP3, Helios, and TIGIT, as well as a high TNFα-producing potential. That depletion of TNFα using Etanercept led to a reduction of Treg-associated markers including FOXP3, Helios, TIGIT, and EZH2 further supports a role for TNFα–TNFR2 signaling in the FOXP3 expression of Treg and the notion of an autocrine TNFα–TNFR2 feedback loop that promotes Treg stability. Consistent with our data, in an acute graft-versus-host disease (aGvHD) mouse model the treatment with a selective TNFR2 agonist led to the *in vivo* expansion of host Treg and the protection from aGvHD (48). Interestingly, the suppressive activity of Treg to control GvHD seems to depend on TNFα produced by donor T cells and TNFR2 expressed on Treg in allogeneic hematopoietic stem cell transplantation (49). In response to TCR stimulation, CD4^+^FOXP3^−^ Teff as well as cytotoxic CD8^+^ cells also upregulates TNFR2 expression. TNFR2-positive CD4 Teff are highly proliferative and more resistant to Treg-mediated inhibition (50). Intriguingly, TNFR2 agonism effectively and selectively induces the apoptosis of insulin-autoreactive CD8^+^ cells in patients with type 1 diabetes (51). Therefore, specific TNFR2 agonism would have two desired cellular immune effects for treatment of autoimmune diseases: (1) selective death of autoreactive T cells and (2) expansion of beneficial Treg. The positive effect of TNF–TNFR2 activation on Treg numbers is also reported in cancer patients. For example, enhanced abundance of TNFR2^+^ Treg and high TNFα serum level were reported in patients with ovarian cancer, lung cancer as well as colorectal cancer (33, 52, 53). In a mouse model of colorectal cancer, blockade of TNFα–TNFR2 signaling prevented rapid resurgence of Treg after cyclophosphamide-induced lymphodepletion and inhibited the growth of established tumors (33).

The effect of TNFα on human Treg is not yet fully clear. Oppenheim and colleagues showed TNF-induced Treg (iTreg) proliferation and survival *via* TNFR2 (25, 47) and TNFR2^+^ Treg exhibited maximal suppressive capacity (29). In the context of autoimmunity, Treg suppressive function is optimized by pathogenic T cells and TNFα is one of factors involved in this optimization (54). Zaragoza et al. reported that TNFα together with IL-2 increased the expression CD25 and FOXP3 and maintained the suppressive activity of human Treg (31). In our cell culture system, we noticed that, upon stimulation with CD28-SA + Rap, the addition of exogenous soluble rhTNFα showed a similar effect as the addition of TNFR2 agonist on the maintenance of a *bona fide* Treg phenotype, whereas blocking TNFα signaling using Etanercept decreased the frequency of FOXP3-positive cells (Figure 3C). This supports the positive effect of TNFα on the Treg phenotype. Previously, TNFα was shown to downregulate Treg function since the TNFα-blocking agent Infliximab increased FOXP3 expression and restored their suppressive function in rheumatoid arthritis (RA) patients (32). However, a follow-up study demonstrated that iTreg, but not naturally occurring Treg (nTreg) were increased in RA patients following anti-TNF therapy (55). Of note, nTreg and iTreg differentially require TNFα signals for optimal suppressive function, at least in mice (28). Nie et al. showed that TNFα impaired Treg suppressive function *via* the dephosphorylation of FOXP3 protein (30). They also demonstrated that rhTNFα did not affect FOXP3 expression; instead, TNFα enhanced the expression of protein phosphatase PP1 which mediated FOXP3 dephosphorylation, thus rending the Treg defective. It is worth noting that anti-TNF therapy often results in psoriatic and lupus-like symptoms in patients being treated for other conditions (56); this suggests a direct correlation between TNF and immune suppression.

Metabolic changes directly modify T cell function. Signaling *via* PI3K–Akt–mTOR pathway facilitates the induction of glucose transporter Glut 1 and aerobic glycolysis in Teff (57). Interestingly, proliferative Treg cells have high mTOR activity as well as high glucose uptake together with downregulated FOXP3 expression and impaired suppressive capacity (58). FOXP3 expression is inversely related to Akt activity (59) and promotes mitochondrial oxidative metabolism. It would seem that Treg proliferation and suppressive function is regulated by separate metabolic pathways. Rapamycin induced retardation of Treg growth might be caused by the shifting of glycolysis metabolism to lipid oxidative metabolism *via* the inhibition of the PI3K–Akt–mTOR pathway and enhanced FOXP3 expression. Non-canonical NFκB activation upon TNFα stimulation is involved in T cell survival and differentiation (23). Here, we showed that the combined addition of rapamycin and TNFR2 agonist resulted in high expression of Treg associated marker, and activation of NFκB pathway. The autocrine feedback loop of TNFα and TNFR2 might fine-tune the metabolic balance between glycolysis and oxidative phosphorylation, thereby favoring homogenous Treg proliferation together with the preservation of potent suppressive function. Further experiments on metabolic pathway regulation are required to test this hypothesis.

Epigenetic mechanisms that alter chromatin organization are important to control the differentiation and maintenance of polarized T cell subsets. EZH2 functions primarily within the polycomb repressive complex 2 and catalyzes the tri-methylation of lysine 27 on the exposed N-terminal tail of histone H3 (H3K27me3), a histone modification associated with repression of expression of nearby genes. EZH2, *via* the formation of a complex with FOXP3 in activated Treg, is crucial for proper Treg suppressive function since mutant mice bearing Treg-specific deletion of EZH2 developed fatal inflammation associated with massive T cell activation and cytokine production (35, 36). Mice that specifically lack EZH2 expression in Treg develop spontaneous inflammatory bowel disease (37), which further supports the crucial role of EZH2 for Treg function. Recently, human Treg were reported to express *EZH2* mRNA (60). In our current study, we demonstrate that CD28 superagonist stimulation induced EZH2 expression in human Treg, which was decreased by the presence of rapamycin, whereas the combined addition of rapamycin and TNFR2 agonist to Treg cultures maintained expression of EZH2 in a TNFα-dependent manner. Interestingly, the NFkB family of proteins RelA as well as c-Rel were reported to enhance luciferase activity in an *EZH2* reporter system, and c-Rel regulated the induction of *EZH2* gene expression in activated primary murine lymphocytes and human leukemia cell lines (61).

In summary, we showed that stimulation of human Treg using a triple combination of CD28 superagonist, rapamycin, and TNFR2 agonist leads to homogenous expansion of Treg that reveal a stable and suppressive phenotype. Mechanistically, rapamycin enhanced TNFR2 expression of the CD28 superagonist-stimulated Treg; the TNFR2 agonist promotes TNFα production and this supports an autocrine TNFα–TNFR2 feedback loop that favors high expression of TIGIT, FOXP3, Helios, and EZH2.

## Ethics Statement

Peripheral blood mononuclear cells (PBMCs) were isolated by density gradient centrifugation (Lymphoprep, Nycomed Pharma AS, Oslo, Norway) of buffy coats that were purchased from Sanquin blood bank (Region South-East, Netherlands). All donors gave written informed consent for the use of these buffy coats for scientific research purposes and according to Dutch law.

## Author Contributions

XH, PU, HK, and IJ designed experiments; XH and PU performed experiments and analyzed the data. XH, PU, HK, and IJ wrote the manuscript. All authors reviewed the manuscript.

## Conflict of Interest Statement

The authors declare that the research was conducted in the absence of any commercial or financial relationships that could be construed as a potential conflict of interest.
