# Supplementary material for: An Autocrine TNFα–Tumor Necrosis Factor Receptor 2 Loop Promotes Epigenetic Effects Inducing Human Treg Stability In Vitro
Source: Front Immunol. 2018 Mar 21;9:573. doi: 10.3389/fimmu.2018.00573 (PMC5871762; doi:10.3389/fimmu.2018.00573)
Supplement: Supplementary file 1 [file data_sheet_1.PDF]

Supplementary Figure S1: Effect of the TNFR2-agonist on cultured Treg. FACS-sorted human Treg were cultured in the presence or absence of TNFR2-agonist (2.5 μg/mL) for 7-days. Thereafter, cells were harvested and stained with proper fluorescence-conjugated mAbs. (A) Percentage of dead cells within the CD4+ cell gate is shown. (B) Percentage and MFI of TNFR2 based on the staining of TNFR2/APC mAb. (C) IL-17A, IFNγ, and TNFα production were analyzed by Luminex.
(D) Percentage of positive cells based on the staining of CD25/Krome Orange, HLA-DR/FITC, FOXP3/eFluor450, Helios/AlexFluor 647, and TIGIT/PE mAbs. Data in the cumulative data graph show the percentage of positive cells. All data are shown as mean +- SEM. N = 4. Wilcoxon matched-pairs signed rank test is used to compare two groups. Asterisks were shown only when $p<0.05$.
TNFR2-ago: TNFR2-agonist; nd: not detected.

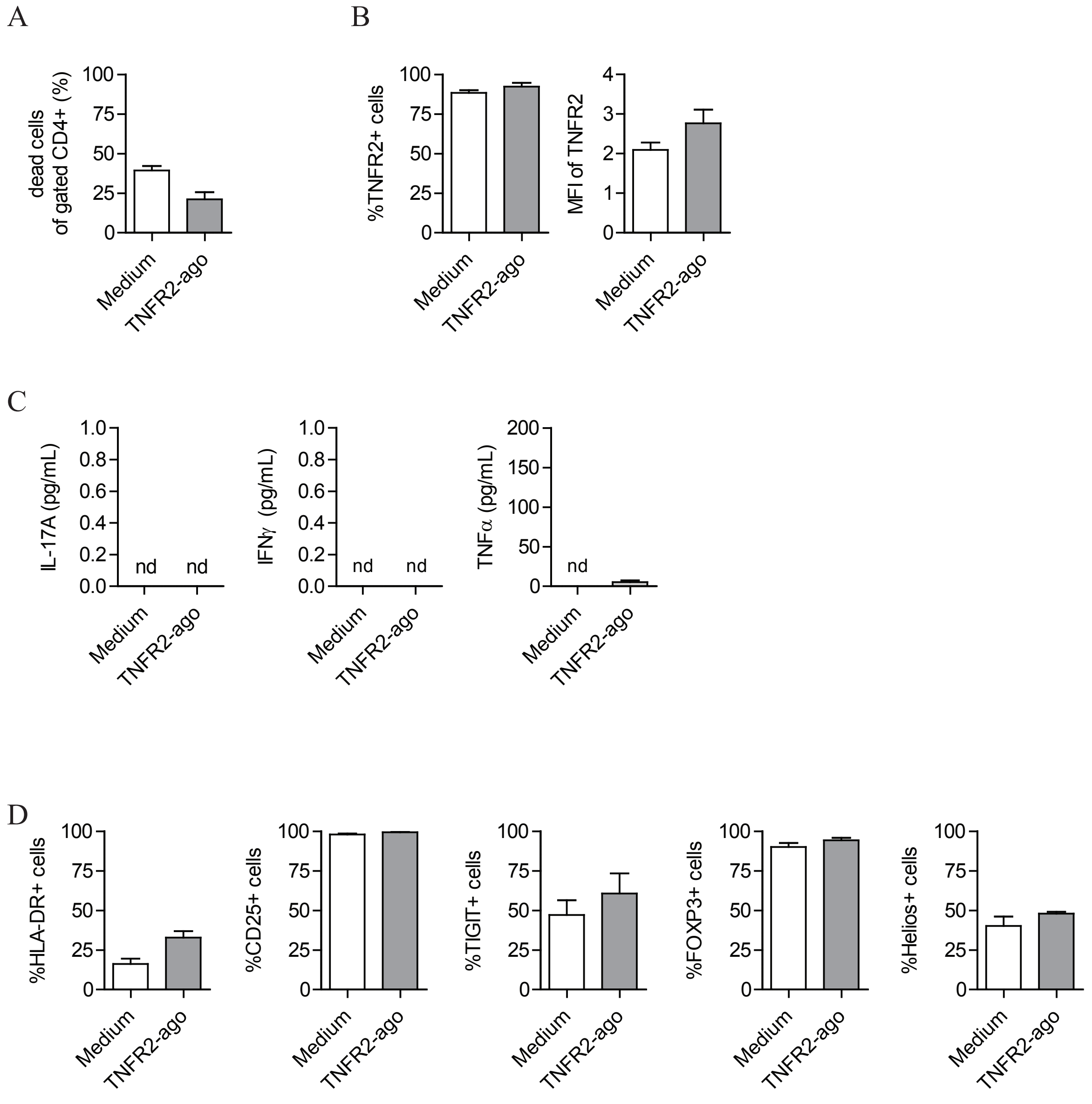

Supplementary Figure S2: Cytotoxic effect of rapamycin and/or TNFR2-agonist on cultured Treg. FACS-sorted human Treg were cultured for 7-days under the different conditions as indicated on the X-axis. Thereafter, cells were harvested and stained with the appropriate fluorescence-conjugated mAbs and Fixable Viability Dye eFluor 780. Percentage of dead cells within the gated CD4+ cells is shown. N = 7.

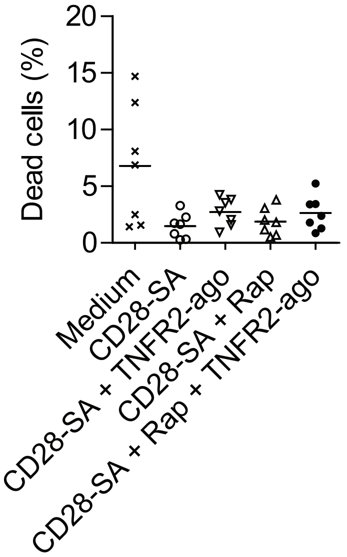

Supplementary Figure S3: Detection of the surface expression of TNFR2 using antibodies derived from different clones. FACS-sorted human Treg were stimulated with CD28-SA + Rap + TNFR2-agonist (Clone MR2-1) for 7-days. At day 7 of culture the expression of TNFR2 was analyzsed using (A) FITC-conjugated anti-TNFR2 mAb (Clone MR2-1) or (B) APC-conjugated anti-TNFR2 mAb (Clone #22235). (C) FACS-sorted human Treg were stimulated using CD28-SA+Rap for 7-days. Thereafter, cells were either stained directly with anti-TNFR2/APC (Clone #22235, black line), or firstly incubated with TNFR2-agonist (Clone MR2-1) for 30 min and thereafter stained with anti-TNFR2/APC (Clone #22235, red line). N = 2.

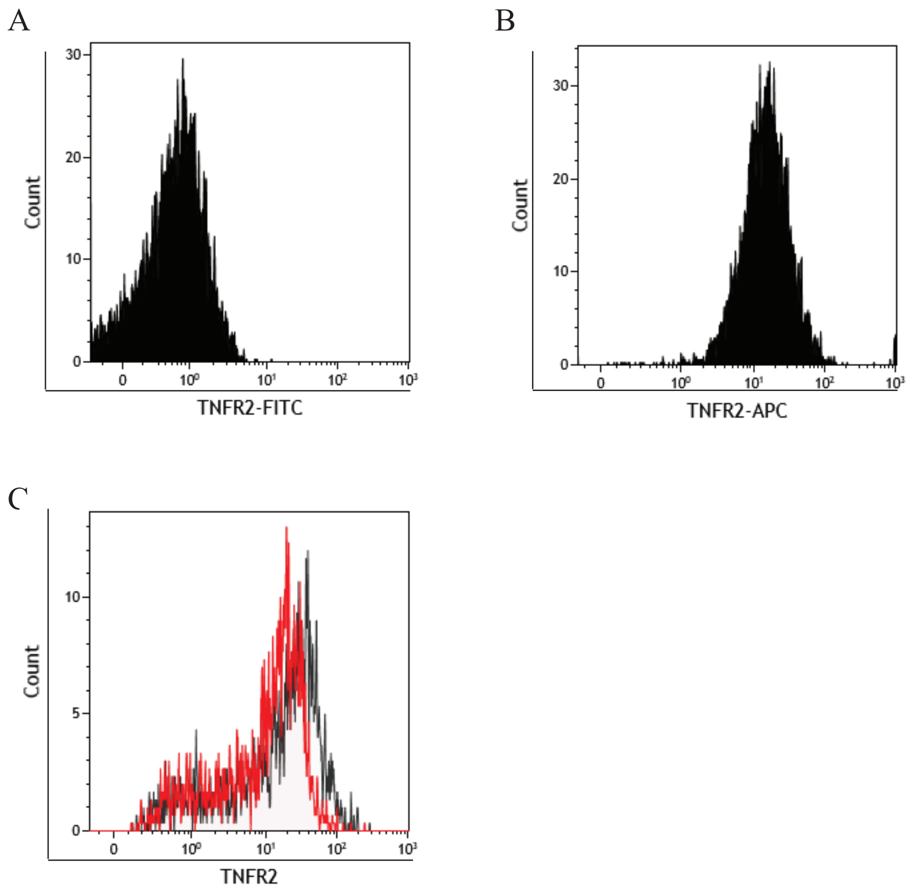

Supplementary Table S1. Primers used for RT-qPCR.

**Code**	**Gene**	**Encoded Protein**
4333768T	*HPRT1*	HPRT
Hs00765730_m1	*NFKB1*	NFκB1/p50
Hs01028901_g1	*NFKB2*	NFκB2/p52
Hs00355671_g1	*NFKBIA*	IκBα
Hs00153294_m1	*RELA*	RelA/p65
Hs00232399_m1	*RELB*	RelB
Hs00174128_m1	*TNFA*	TNFα
Hs00961750_m1	*TNFRSF1B*	TNFR2
